# Age-related deterioration of performance and increase of cortex activity comparing time- versus item-controlled fNIRS measurement

**DOI:** 10.1038/s41598-021-85762-w

**Published:** 2021-03-24

**Authors:** Leonore Blum, David Rosenbaum, Benjamin Röben, Katja Dehnen, Walter Maetzler, Ulrike Suenkel, Andreas J. Fallgatter, Ann-Christine Ehlis, Florian G. Metzger

**Affiliations:** 1 Department of Psychiatry and Psychotherapy, University Hospital of Tuebingen, Tuebingen, Germany; 2 German Center for Neurodegenerative Diseases (DZNE), University of Tuebingen, Tuebingen, Germany; 3 Department of Neurodegenerative Diseases, Hertie Institute for Clinical Brain Research, University Hospital Tuebingen, Tuebingen, Germany; 4 Geriatric Center, University Hospital of Tuebingen, Tuebingen, Germany; 5 LEAD Graduate School and Research Network, University of Tuebingen, Tuebingen, Germany; 6 Department of Neurology, Christian-Albrechts-University, Kiel, ,Kiel, Germany; 7 Institute for General Medicine, University Hospital of Essen, Essen, Germany; 8 Vitos Hospital of Psychiatry and Psychotherapy Haina, Haina, Germany

**Keywords:** Medical research, Neurology, Risk factors

## Abstract

In our aging society, research into neurodegenerative processes is of great interest. Thereby, cortical activation under different neurocognitive conditions is considered to be a promising predictor. Against this background, the executive functions of a total of 250 healthy older adults (53–84 years) have been investigated using the Trail Making Test (TMT) and functional near-infrared spectroscopy in a block design. We investigated effects of age on the performance and cortical blood oxygenation during the TMT. Since it is assumed that older people may compensate for cognitive deficits by slowing their processing speed, we additionally analyzed the cortical blood oxygenation per solved item. Our results showed a significant decrease in processing speed in older participants compared to middle-aged individuals, however, also lower error rates during TMT part A. On a neurophysiological level, we observed increased cortical blood oxygenation in the older participants when completing the TMT. Finally, with respect to the combined measurement (O_2_Hb/item), no significantly higher hemodynamic cortical response per item was found within the older participants. The results confirm a deterioration of cognitive performance and an increase of cortical activity with increasing age. The findings are discussed in the light of current research.

## Introduction

The demographic development in Europe tends towards an ageing population due to an increasing life expectancy, caused by more prosperity, better medical care and a decline in the birth rate. Over the next two decades, the current number of people over 65 years of age is expected to double^1^. Age is regarded as the greatest risk factor for the development of neurodegenerative diseases such as Parkinson´s disease (PD) and Alzheimer´s disease (AD). Currently, the worldwide prevalence of dementia is around 46 million. This is expected to rise to 132 million by 2050^2^ but the underlying mechanisms are still not sufficiently researched.

The brain, which is the affected organ in neurodegenerative diseases, is subject to lifelong structural^3^ and functional change^4^. Age-dependent processes of brain structure seem to make tissue more susceptible to neurodegenerative diseases^5^. In MRI studies, an age-dependent decrease in grey and white matter of 0.5–1% per year was observed^6–9^. This phenomenon can be explained by a decrease in neuronal volume and synaptic branching^3^. Affected are mainly the prefrontal cortex (PFC) and hippocampus brain regions, which play an essential role in learning and memory storage^10^. The decline in cognitive ability in old age primarily affects working and episodic memory, resulting in reduced processing speed and decreased mental flexibility^11^. In addition, deficits in decision making and speech processing are mentioned^12^. Against this background, it is essential to be able to detect neurodegenerative processes that lead to a decline in cognitive function at an early stage.

Functional near-infrared spectroscopy (fNIRS), a neurophysiological measurement technique that is based on the detection of hemodynamic changes in cortical oxygenated and deoxygenated blood levels that follow neuronal activity^13^, combines decisive advantages, such as investigations under ecologically valid conditions (e. g. tests in realistic environment instead of a narrow tube (MRI), social interaction, investigations in an upright sitting position) and insensitivity to movement artifacts^14^.

In this study, the advantages of fNIRS were used to measure the cognitive abilities of a large group of participants under realistic and physiological conditions using the Trail Making Test (TMT). The TMT is used to assess cognitive performance. It facilitates the assessment of various neuropsychological parameters such as mental flexibility, working memory, visuomotor processing speed and executive functions^15^. Classically, the TMT is divided into parts A and B, but there is also part C in some versions of the TMT. In the TMT-A, the subject is asked to connect numbers on a sheet of paper in ascending order with a pencil as quickly as possible. Both TMT versions (A and B) require visual search function, motor speed abilities, and recall of working memory. The TMT-B additionally assesses the ability of task-switching by requiring participants to alternately connect numbers and letters in ascending order of the number chain/alphabet^16^. The TMT is usually evaluated by measuring the time to completion^17^. If the respondent makes a mistake, he or she is immediately told that he or she made a mistake and pointed back to the last correct item. The time measurement is not interrupted during this time^18^. TMT-A is considered a simpler task. For this reason, higher activation during TMT-B can be expected in brain regions recruited for both tasks. Contrary to these expectations, this effect has not been reported consistently in the literature, especially when block designs are used^4^. One possible explanation is that both, processing speed and task complexity, play a role in the level of blood oxygenation^15,19^.

In one of our previous studies, we found that task complexity and processing speed both have effects on cortical blood oxygenation, implicating that results from TMT paradigms in older individuals might be compromised if the participants slow down speed to compensate for age-related deficits^19^. Therefore, we suggested to either use a TMT paradigm in which different speed levels are realized or to use an additional dependent variable: blood oxygenation per solved item. The correction method, developed in our first study, relates averaged performance and individual blood oxygenation per item (relative O_2_Hb values per processed item within the block ((mmol x mm)/item). The validity of this simplified correction method was tested by a parallel regression calculation with analysis of the participants’ individual O_2_Hb values after regressing out the number of processed items for all TMT conditions in three different speed levels.

Regarding behavioral effects, the comparison with previous literature confirmed a deterioration in performance during the TMT with increasing age^20,21^, with both an increase in processing time^22,23^ and a decrease in accuracy^20,24^. A study by Rodewald et al^25^. compared the TMT processing times of different age groups and found an age effect, characterized by reduced processing speed for the TMT-B in older participants. A longitudinal study by Rasmusson et al.^23^ revealed similar results confirming increased completion times for both TMT-A and TMT-B for older participants. While the processing time for TMT-A remained unchanged, a significant increase for the TMT-B time within the two-year period was found.

On a neurophysiological level, various studies agree that older adults show higher cortical activation than younger adults when performing demanding tasks^26–28^, which has often been interpreted as evidence of a compensatory mechanism. fNIRS studies, for example, by Herrmann et al.^29^ and Muller et al.^30^ detected altered activation patterns in older participants with more bilateral activation in the dorsolateral prefrontal cortex (DLPFC) as compared to younger individuals. Apart from the bilateral reorganization, a correlation between overactive brain regions and performance success was noticed in the elderly^31^. A well-known model that incorporates the idea of a compensation mechanism is the Compensation-Related Utilization of Neural Circuits Hypothesis (CRUNCH)—model^32^. It assumes that older adults respond to increasing cognitive task severity with additional recruitment and activation of brain regions. Since older individuals reach their load limit earlier than younger persons, an increase in brain activation in older individuals can already be expected in the lower/medium task load in the sense of a classical compensation. However, the additional possible recruitment is limited, which is why older adults in the higher performance range eventually show a worsened performance and lower activation compared to the younger group^32^. Another frequently discussed hypothesis to the age-related decline in cognitive performance is the neural dedifferentiation^26,33,34^. The hypothesis implies a decrease in neural differentiation with age, accompanied by lower selectivity in neural processing and functional specificity of individual brain regions^33,35^.

In light of the aforementioned cognitive aging models, our study aims to contribute to a better understanding of the interaction of neural activations and performance in older age by comparing two different approaches: First, the cortical blood oxygenation (O_2_Hb during task completion; time-controlled) and second, as an additional index, the item-controlled blood oxygenation (O_2_Hb/item). In addition, we investigated the effects of age on behavioral performance during the TMT (solved items). We assumed an age-related reduction of processing speed, as well as an increased hemodynamic response during the TMT. Furthermore, by comparing the classical time-corrected results with the results of a within-subject correction method for the number of processed items, we expected to highlight influences of performance on brain activation.

## Material and methods

### Study design

Data have been collected during the TREND study at the Department of Psychiatry and Psychotherapy of the University Hospital of Tuebingen, Germany which started in 2009. Since then, a total of 1201 volunteers have been examined every 2 years. The aim of the TREND study is to investigate possible prodromal markers for neurodegenerative diseases. After a detailed anamnesis, the participants complete a circle of test stations with several neurological individual examinations. As part of the Consortium to Establish a Registry for Alzheimer`s Disease (CERAD) Plus test battery, brain activity was measured by functional near-infrared spectroscopy (fNIRS) while the participants completed the Trail Making Test (TMT). The cohort is composed of 250 older adults, covering an age range from 53 to 84 years. The available data were collected during the second follow-up in spring 2013—autumn 2014 and represent a cross-sectional study. The study design was reviewed and approved by the Ethics Committee at the University Hospital and University of Tuebingen and complies with the standards of the declaration of Helsinki in its latest version. Autonomous informed consent was obtained from all participants.

### Participants

In the current study we extracted a sample of 125 older participants (> 66 years old) and matched a group of 125 younger participants (< 66 years old) according to gender and neurodegenerative pre-existing conditions. The younger group showed an average age of 59.94 (*SD* = 3.60) years and 14.43 (*SD* = 2.52) years of education. Within the older cohort the mean age was 72.27 (*SD* = 4.58) years with an average of 14.24 (*SD* = 2.78) years of education. The groups did not differ in terms of gender distribution (χ^2^(1) = 0.41, *p* = 0.520) or years of education (*t*(248) = 0.57, *p* = 0.568, d = 0.072) and neurodegenerative pre-existing conditions (amnestic Mild Cognitive Impairment (aMCI) (χ^2^(1) = 1.36, *p* = 0.243); REM sleep behavior disorder (RBD) (χ^2^(1) = 0.03, *p* = 0.860).

Within the whole sample of 250 participants, 86% took medication: Mainly blood pressure medication (42%), anticoagulants (26%) and antidepressants (10%). The age groups differed concerning the blood pressure medication (χ^2^(1) = 912.91, *p* = 0.000) (53% within the older group, 30% within the younger group took blood pressure medication) and anticoagulants/antiplatelet drugs (χ^2^(1) = 26.68, *p* = 0.000) (41% within the older group, 12% within the younger group took anticoagulants/antiplatelets). Concerning the antidepressants (χ^2^(1) = 1.659, *p* = 0.198) (7% within the older group, 12% within the younger group took antidepressants) no differences were detectable.

The medication was recorded independently of the underlying diagnosis, since an influence of medication on the fNIRS signal cannot be excluded.

### Experimental setup

#### Trail making test

The measurement was performed in a quiet darkened room in order to reduce the influence of light or auditory stimuli on the measurement results. The participants were instructed to take an upright sitting position and to avoid head movements as far as possible, since a displacement of the optodes and movement artifacts should be prevented. A DIN A4 worksheet was attached to an inclined desk to ensure physiological operation. After attaching the fNIRS-optodes, the participants received a detailed working instruction and a pencil. They were instructed to complete the test as quickly as possible “without lifting the pen” as described in the CERAD_Plus test battery. Each version of the TMT consists of 25 items.

The procedure started with a five-minute resting-state measurement with closed eyes. Then the TMT started in the order TMT-C, TMT-A, TMT-B, which was repeated once. Each TMT measurement began with a 10 s baseline measurement with open eyes; afterwards the TMT was performed for 30 s and then stopped. TMT-C was used as a modification of the usual TMT to assess cortical activation solely related to motor skills by requiring the participants to trace dotted lines (control condition). The number of processed items and errors was documented.

#### Functional near-infrared spectroscopy

In the current study, brain activation was detected by a multichannel fNIRS system (ETG-4000 Optical Topography System; Hitachi Medical Co., Japan). Multiple optodes of two wavelengths (695 nm and 830 nm) measure oxy- and deoxyhemoglobin simultaneously with a sampling rate of 10 Hz.

**Figure 1:**
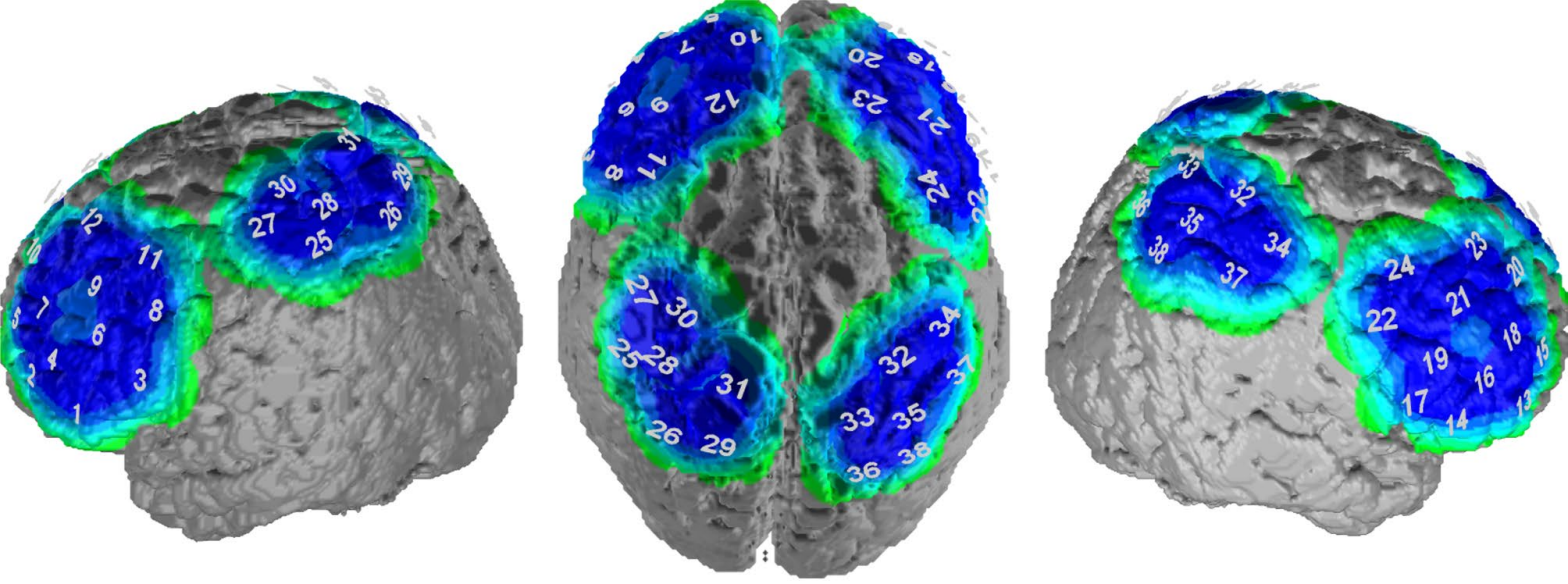
Probeset coordinates.

Three probesets (1A, 1B, 2) with 38 channels were fixed on the volunteer’s head using an optode holder cap: One parietal (14 channels) and two covering left and right fronto-temporal areas (12 channels each; see Fig. 1). The exact placement of the optode holder cap on the skull was controlled by the reference points Fpz and Cz according to the international 10–20 system. The arrangement of the channels to corresponding brain regions was based on the Colin template^36–38^.

### Data analysis

The measured fNIRS signals were processed and visualized with the ETG-4000 system software. The data set was then exported (CSV-file format) and analyzed with MATLAB R2017a (MathWorks Inc., Natick, USA).

Data on cortical blood oxygenation in terms of oxygenated (O_2_Hb) and deoxygenated hemoglobin (HHb) were computed by means of a modified Beer–Lambert-Law. Preprocessing included correction of high amplitude artifacts by the TDDR correction^39^, bandpass filtering (0.01–0.1 Hz), and a correlation based signal improvement described by Cui et al.^40^. The following steps included a visual inspection of the data and interpolation of strongly deviating outlier channels by proximate channels as well as a correction for the global signal changes by a PCA-based kernel filter^41^. Furthermore, data were individually averaged across block repetitions with a baseline correction to eliminate baseline drifts. Additionally to O_2_Hb levels, we computed an item-controlled within-subject correction by dividing the fNIRS O_2_Hb values by the number of completed items, generating the additional variable: O_2_Hb/processed items ((mmol x mm)/item)^19^.

After pre-processing, fNIRS data were further analyzed with IBM SPSS Statistics Version 24. The analysis of behavioral data was calculated by a two (age: younger vs. older participants) by three (TMT: TMT-C vs. TMT-A. vs. TMT-B) repeated measures ANOVA. For post-hoc analysis Helmert contrasts (TMT-C versus TMT-A/B, TMT-A versus TMT-B) were used. To account for the missing normal distribution, number of errors committed during the TMT was compared between the two age groups by using non-parametric Mann–Whitney tests. To analyze fNIRS data, we performed repeated measurement MANOVAS with the factors age (< 66 and ≥ 66; between-subjects) and TMT (levels A, B, C; within-subjects), for the depended variables of each ROI (left and right dorsolateral prefrontal cortex (left DLPFC, right DLPFC), left and right inferior frontal gyrus (left IFG, right IFG), left and right somatosensory association cortex (left SAC, right SAC). In case of significant effects in the MANOVA, we investigated univariate statistics and corrected for multiple comparisons by the Benjamini–Hochberg procedure. Further, we repeated the analysis with item-corrected data.

Finally, we explored on how far the association between the factor of age and the dependent variables (fNIRS data, completed items) is linear or quadratic. For this aim, we computed mixed level models in which we regressed age and the quadratic age term in separate models with random intercepts on the dependent variables (items, left DLPFC, right DLPFC, left IFG, right IFG, left SAC, right SAC).

## Results

### Behavioral performance

On a behavioral level, a two (age: younger vs. older participants) by three (TMT: TMT-C vs. TMT-A. vs. TMT-B) ANOVA showed a significant main effect of TMT (*F*(1.95, 477.99) = 1536.91, *p* = 0.000, partial η^2^ = 0.86) and a main effect of age (*F*(1, 245) = 37.64, *p* = 0.000, partial η^2^ = 0.13). Post hoc analysis revealed fewer processed items during TMT-A/TMT-B in comparison to TMT-C (*F*(1, 245) = 1568.75, *p* = 0.000, partial η^2^ = 0.87), fewer processed items during TMT-B in comparison to TMT-A (*F*(1, 245) = 1511.33, *p* = 0.000, partial η^2^ = 0.86) and fewer processed items in older than in younger participants (TMT-A: *t*(211.73) = 6.13, *p* = 0.000, d = 0.78), (TMT-B: *t*(240.33) = 4.45, *p* = 0.000, d = 0.56) (Table 1). Male and female participants did not differ in terms of the number of processed items (TMT-A: *t*(248) = 4.16, *p* = 0.677, d = 0.05), (TMT-B: *t*(248) = 1.41, *p* = 0.161, d = 0.18).

**Table 1:** Number of processed items and errors during TMT-A, TMT-B and TMT-C, depending on age.

		Middle-aged participants (< 66 years)		Older participants (> 66 years)	
		Mean	*SD*	Mean	*SD*
TMT-A	Processed items	22.31	3.14	19.14	4.87
TMT-B	Processed items	11.50	4.09	9.28	3.41
TMT-C	Processed items	23.86	0.85	23.57	1.49
TMT-A	Mean error rates	0.17	0.31	0.08	0.21
TMT-B	Mean error rates	0.22	0.49	0.16	0.36
TMT-C	Mean error rates	0.01	0.06	0.03	0.13

In addition, we found an interaction of age by TMT (*F*(1.95, 477.99) = 16.22, *p* = 0.000, partial η^2^ = 0.06) reflecting that the age groups showed significant differences during TMT-A/B in comparison to TMT-C (*F*(1, 245) = 30.45, *p* = 0.000, partial η^2^ = 0.11) as well as for the contrast of TMT-A and TMT-B (*F*(1, 245) = 4.80, *p* = 0.029, partial η^2^ = 0.02). As indicated by these results, age and number of processed items were negatively correlated (TMT-A: *r*(248) = −0.50, *p* = 0.000), (TMT-B: *r*(248) = −0.40, *p* = 0.000).

With respect to error rates, we found a significantly reduced error rate in the older participants (mean = 0.08 errors (*SD* = 0.21) in comparison to the younger participants (mean = 0.19 errors (*SD* = 0.31) during TMT-A (*U* = 6780.50, *z* = −2.63, *p* = 0.008, *r* = −0.17) (Table 1). However, groups did not differ in error rates during TMT-B and TMT-C. A within-subject analysis of the older participants found no correlation between the number of completed items and the number of committed errors for TMT-A: *r*(125) = −0.04, *p* = 0.628). Furthermore, there were no differences in error rates between men and women (TMT-A: (*U* = 6933.00, *z* = −1.93, *p* = 0.053, *r* = −0.12), (TMT-B: *U* = 7213.00, *z* = −1.11, *p* = 0.265, *r* = −0.07). In total, error rates were very low (TMT-C = 0.02, mean TMT-A = 0.12, TMT-B = 0.19) and most participants performed without errors at all (TMT-C = 97%, TMT-A = 81%, TMT-B = 76%). For this reason, we did not include error rates in the evaluation of item-corrected NIRS data.

### fNIRS Time-Corrected Data

Not surprisingly, a repeated measures MANOVA determined that mean blood oxygenation levels showed a statistically significant difference between the different TMT conditions (TMT-A, TMT-B, TMT-C) (*F*(12, 237) = 2.51, Wilk’s Λ = 0.89, *p* = 0.004, partial η^2^ = 0.11). Further, we observed a main effect for age (younger vs. older participants) (*F*(6, 243) = 2.69, Wilk’s Λ = 0.94, *p* = 0.015, partial η^2^ = 0.06).

**Figure 2:**
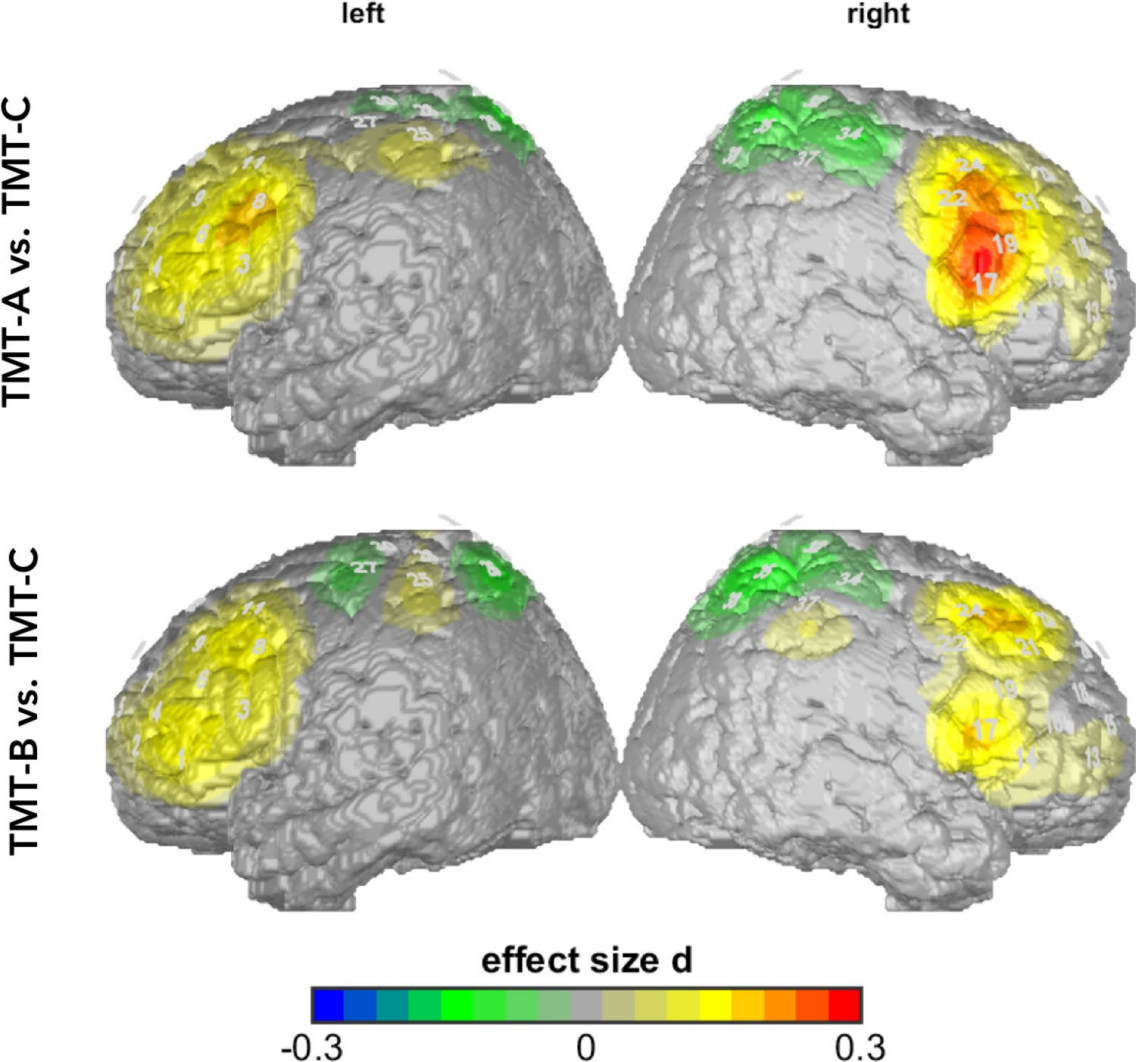
Time-corrected data (uncorrected O_2_Hb values): activity in TMT-A and TMT-B versus TMT-C. Higher activity in TMT-A and TMT-B compared to TMT-C. Differences are shown in effect size Cohen’s d.

The univariate comparison of the main effect for TMT showed significantly deviating O_2_Hb levels in left IFG (*F*(2, 496) = 7.02, *p* = 0.001, partial η^2^ = 0.028) and right IFG (*F*(1.94, 482.23) = 4.46, *p* = 0.013, partial η^2^ = 0.02) and tendencies in the right DLPFC (*F(*1.79, 443.14) = 3.76, *p* = 0.028, partial η^2^ = 0.02) and left DLPFC (*F*(1.92,475.26) = 3.35, *p* = 0.038, partial η^2^ = 0.01). Post-hoc analysis revealed differences between TMT-C contrasted to TMT-A/B in left and right IFG and in left and right DLPFC: left IFG: (*F*(1, 248) = 12.94, *p* = 0.000, partial η^2^ = 0.05); right IFG: (*F*(1, 248) = 8.80, *p* = 0.003, partial η^2^ = 0.03), left DLPFC (*F*(1, 248) = 5.92, *p* = 0.016, partial η^2^ = 0.02), right DLPFC (*F*(1, 248) = 7.02, *p* = 0.009, partial η^2^ = 0.03) (Fig. 2). No differences were observed between TMT-A and TMT-B (all *p* > 0.05).

**Figure 3:**
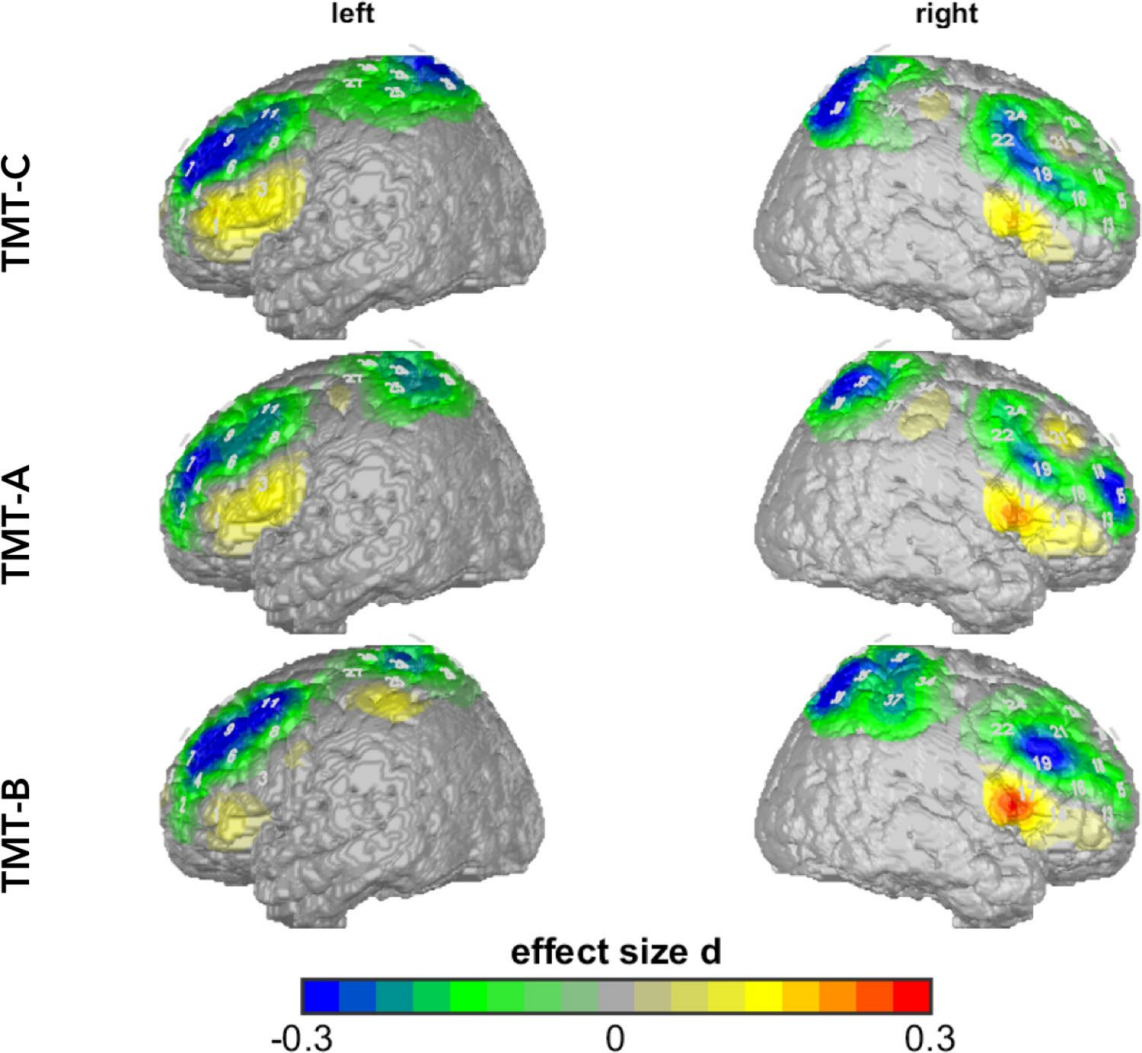
Time-corrected data (uncorrected O_2_Hb values): age effects (participants age < 66 vs. participants age ≥ 66) in TMT-A, TMT-B and TMT-C. Older participants showed higher activity compared to younger participants. Differences are shown in effect size Cohen’s d.

The univariate analysis of the main effect of age specified significant effects in left DLPFC (*F*(1, 248) = 8.77, *p* = 0.003, partial η^2^ = 0.03), right SAC (*F*(1, 248) = 10.00, *p* = 0.002, partial η^2^ = 0.04) and tendencies in the left SAC (*F*(1, 248) = 4.99, *p* = 0.026, partial η^2^ = 0.02) and was characterized by higher O_2_Hb levels in older as compared to younger participants (Fig. 3).

### fNIRS Item-Corrected Data

When data were corrected for the number of processed items, 13 multivariate outliers were found, as assessed by the Mahalanobis distance (*p* > 0.001) and excluded from the analysis. 30.8% of the 13 excluded participants belonged to the younger group and 69.2% to the older group, the sex ratio was 46.2% male and 53.8% female.

As with the time-corrected data, a significant main effect for TMT (*F*(12, 224) = 6.07, Wilk’s Λ = 0.76, *p* = 0.000, partial η^2^ = 0.25) was observed in the item-corrected analysis.

**Figure 4:**
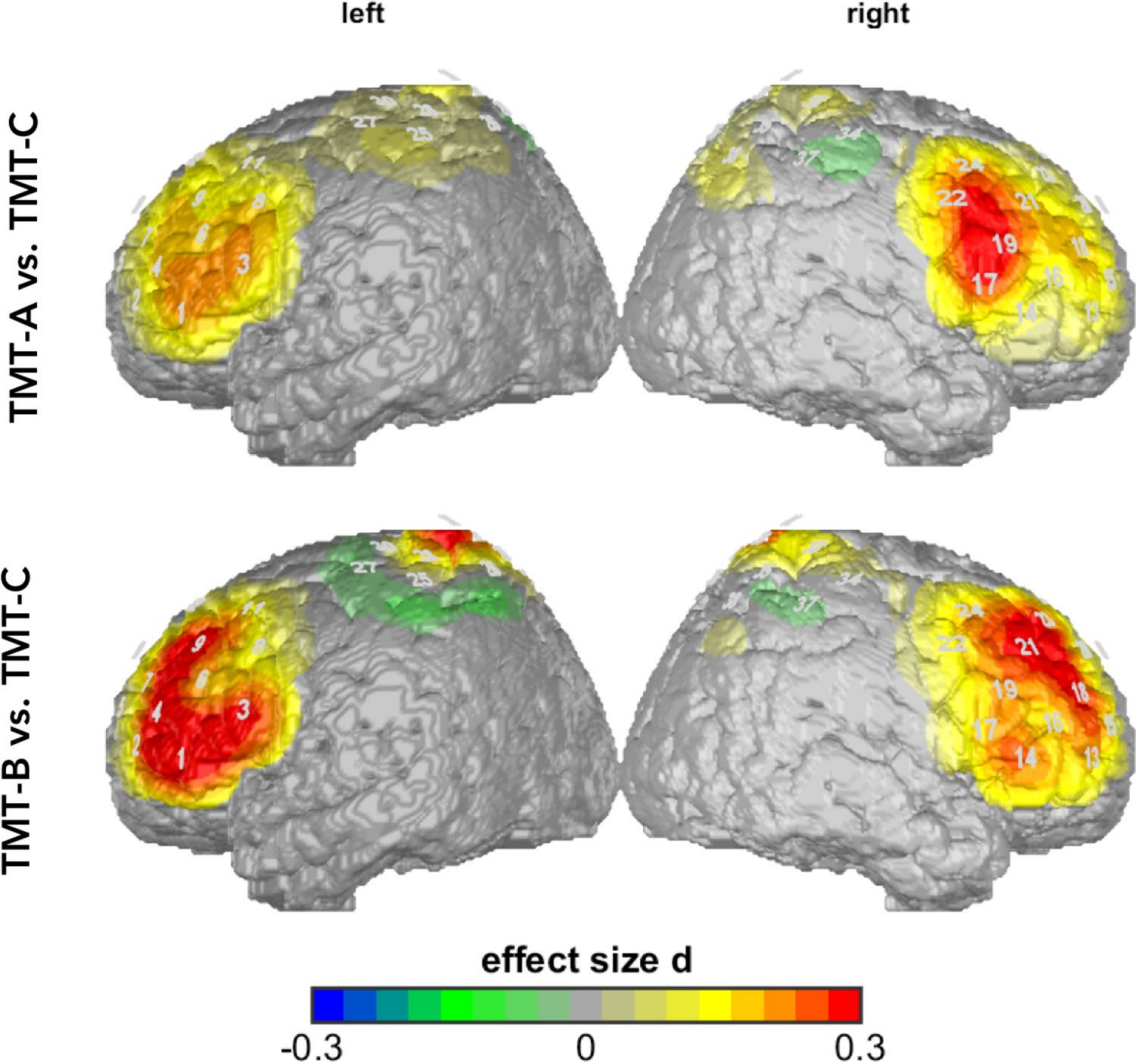
Item-corrected data (O_2_Hb/item): activity in TMT-A and TMT-B versus TMT-C. Differences are shown in effect size Cohen’s d.

The main effect for TMT in the univariate analysis showed up in four ROIs: left IFG (*F*(1.34, 314.48) = 36.40, *p* = 0.000, partial η^2^ = 0.13), right IFG (*F*(1.28, 301.23) = 6.74, *p* = 0.006, partial η^2^ = 0.03), left DLPFC (*F*(1.18, 277.91) = 8.15, *p* = 0.003, partial η^2^ = 0.03), right DLPFC (*F*(1.18, 277.35) = 10.47, *p* = 0.001, partial η^2^ = 0.04) (Fig. 4). Post-hoc analysis of item-corrected data indicated significant differences between TMT conditions for the first Helmert contrast (TMT-C vs. TMT-A/B) as well as for the second Helmert contrast (TMT-A vs. TMT-B). With respect to the first Helmert contrast we observed differences in four ROIs: lDLPFC: (*F*(1, 235) = 14.23, *p* = 0.000, partial η^2^ = 0.06); rDLPFC: (*F*(1, 235) = 18.59, *p* = 0.000, partial η^2^ = 0.07); lIFG: (*F*(1, 235) = 52.10, *p* = 0.000, partial η^2^ = 0.18) and rIFG: (*F*(1, 235) = 17.56, *p* = 0.000, partial η^2^ = 0.07). Further, through the item-correction, effects for the second Helmert contrast (TMT-A vs. TMT-B) became also visible: left IFG: (*F*(1, 235) = 28.59, *p* = 0.000, partial η^2^ = 0.11) and right DLPFC (*F*(1, 235) = 6.13, *p* = 0.014, partial η^2^ = 0.03). The effect indicates higher cortical blood oxygenation per item during TMT-B in comparison to TMT-A.

No between-subjects effect for age was seen in the item-corrected data (*F*(6, 230) = 1.41, Wilk’s Λ = 0.97, *p* = 0.212, partial η^2^ = 0.04).

### Exploratory analysis

The results of our item corrected analysis raised the question of why the effect of age was no longer significant, as older individuals generally showed higher brain activity and fewer completed items. Therefore, we explored the association of age, processed items and O_2_Hb levels in greater detail.

**Figure 5:**
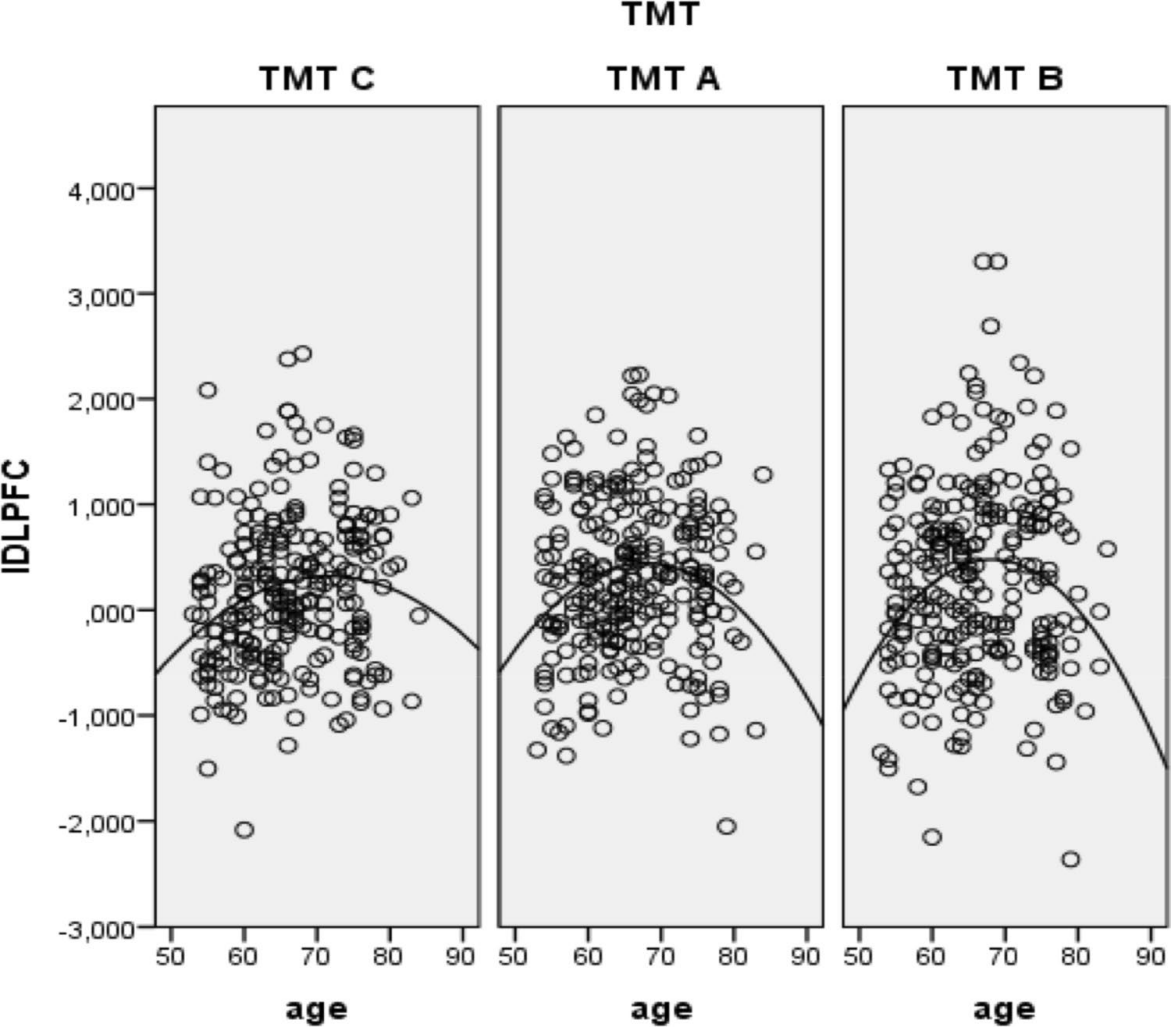
Negative quadratic relationship between age and fNIRS data in left DLPFC.

Interestingly, while the analysis indicated a linear negative relationship between age and completed items, we observed a negative quadratic relationship between age and the left DLPFC and right SAC (Table 2 and Fig. 5/6). This negative quadratic relationship showed a turning point between 65 to 70 years and was characterized by relative increases in O_2_Hb levels up to 65 to 70 years and decreases in O_2_Hb levels with increasing ages above 70 years.

**Table 2:** Results of the mixed models on the polynomic relationship between age and the DVs.

	Model 1	Model 2	
DV	Age	Age	Age^2^
Items	t_(745)_ = 14.07 ***p < .001*** β = −.18	t_(744)_ = 0.98 *p* > .1	t_(744)_ = 0.32 *p* > .1
Left IFG	t_(248)_ = −.55 *p* > .1	t_(247)_ = 1.25 *p* > .1	t_(247)_ = −1.28 *p* > .1
Right IFG	t_(248)_ = −.85 *p* > .1	t_(247)_ = .14 *p* > .1	t_(247)_ = −.19 *p* > .1
Left DLPFC	t_(247)_ = 1.76 ***p < .1*** β = 0.01	t_(247)_ = 3.62 ***p < .001*** β = 0.35	t_(247)_ = −3.52 ***p < .001*** β = −0.00
Right DLPFC	t_(248)_ = .65 *p* > .1	t_(247)_ = 1.92 ***p < .1*** β = 0.17	t_(247)_ = −1.88 ***p < .1*** β = -0.001
Left SAC	t_(248)_ = 1.72 ***p < .1*** β = 0.01	t_(247)_ = 1.89 ***p < .1*** β = 0.09	t_(247)_ = −1.79 ***p < .1*** β = −0.00
Right SAC	t_(248)_ = 3.26 ***p < .001*** β = 0.02	t_(247)_ = 2.16 ***p < .05*** β = 0.18	t_(247)_ = −1.98 ***p < .05*** β = −0.00

### Controlling for confounders

To determine the influence of medication, we additionally calculated MANCOVAs with the factors blood pressure medication and anticoagulants/antiplatelet drugs as covariates. Interestingly, the results of the time-corrected data remained unchanged (differences in blood oxygenation between the three TMT conditions TMT-A, TMT-B, TMT-C (*F*(12, 235) = 2.38, Wilk’s Λ = 0.89, *p* = 0.006, partial η^2^ = 0.11)) and (differences in blood oxygenation between the two age groups (*F*(6, 241) = 3.35, Wilk’s Λ = 0.92, *p* = 0.003, partial η^2^ = 0.08)), whereas the item-corrected analysis showed an additional age effect between the groups (younger versus older participants (*F*(6, 241) = 2.86, Wilk’s Λ = 0.93, *p* = 0.01, partial η^2^ = 0.07)).

## Discussion

The aim of the study was to investigate the effects of age on performance and cortical blood oxygenation during the completion of a cognitively demanding task. As aging is associated with compensatory developmental effects, we investigated the use of a within-subject correction for the number of completed items to determine an influence of performance on the activity pattern in the TMT in order to accentuate age-related changes.

With respect to the behavioral analysis, our results showed main effects of TMT and age that were characterized by fewer processed items during TMT-B in comparison to TMT-A and fewer processed items in participants over 66 years of age as compared to the younger group. Moreover, an interaction effect of age and TMT revealed significant differences between the age groups for the first (TMT-A/B versus TMT-C) and second (TMT-A versus TMT-B) Helmert contrast. Concerning the error rates, a reduced number of errors was observed for the TMT-A in older participants.

Regarding cortical blood oxygenation levels, a main effect for TMT was detected showing higher O_2_Hb levels during TMT-A/B in comparison to TMT-C. Moreover, a main effect for age was observed in various ROIs, revealing higher O_2_Hb levels for the older group.

When data were corrected for the number of processed items (O_2_Hb/item), the main effect for TMT was more pronounced than in the time-corrected analysis and manifested itself in four ROIs (left IFG, right IFG, left DLPFC, right DLPFC). While the time-corrected measurement only showed TMT effects for TMT-C contrasted to TMT-A/B, the item-correction additionally revealed significant second Helmert contrasts for TMT-A versus TMT-B. Contrary to the time-correction, the item-corrected data showed no age-related effect on brain activity during TMT performance.

Our results confirm previous research findings showing fewer completed items during the TMT in older volunteers^20,22,23,25,30^. Several studies have shown that the execution time of the TMT increases with age, especially for TMT-B^22,23,25^. This fact was interpreted as age-related deterioration of mental flexibility, processing speed and attention^20,42^. Moreover, our results support previous findings of more completed items during TMT-A compared to TMT-B regardless of age^30,43^. This is not surprising since TMT-B is considered the more difficult task requiring further cognitive resources as inhibition and set-shifting. For this reason, an increased age effect in TMT-B seems obvious.

Note that no higher error rate was observed in older age. Instead, we observed a reduced error rate in the older participants during TMT-A. Irrespective of the age group, the number of errors during TMT-A and TMT-C was lower than during TMT-B, confirming a higher degree of difficulty of the TMT-B. It could be argued that the reduction of the processing speed in old age explains a lower error rate in TMT-A, since cognitive deficits are compensated by slowing down according to a shift of the speed-accuracy trade-off^44^. However, in our data this hypothesis was not verified by a correlation between processing speed and error rates within older adults, a fact that could be due to the generally very low error rate. A study by Muller et al.^30^ could not determine any age effects with regard to the number of errors.

The current state of research assumes that TMT performance decreases with age, due to reduced processing speed and accuracy^20–24^. A frequently discussed hypothesis is the compensation of age-related deficits by increasing activity and plastic reorganization of brain regions. According to previous research, an increasing bilateral activation^30^ could be determined in high-performing older participants^28^. It was shown that overactive regions in old age are responsible for performance success^31^, indicating increased cortical resources needed to solve a task. A study by Hagen et al.^4^ was able to determine a higher activity combined with a reduced performance in the right Broca’s area and primary somatosensory cortex as well as in the left primary motor cortex in older participants. In line with our results, other studies report a frontal over-recruitment in old age^45,46^ to maintain executive functions.

As expected, our results showed a major effect for TMT in both the time-corrected and item-corrected version. Remarkably, however, the correction for processed items seems to highlight the effects between TMT conditions. Only the item-correction revealed differences between the hemodynamic response of TMT-A and TMT-B. Regarding this point, the results of previous studies are ambivalent. Whereas some studies found significant effects for TMT with a higher activation for TMT-B than for TMT-A^47,48^, in some time-corrected test approaches, this TMT-effect could not always or only weakly be determined so far^4,30^. This observation was the basis for our previous study on the effects of speed and task complexity^19^. We assumed, since TMTA and TMTB differ in two dimensions – the complexity of the items and processed items, both aspects could have opposing effects on the hemodynamic response. In the original neurophysiological TMT experimental setup, the completion time is used as a primary performance metric. The goal is to complete both TMT subtasks as fast as possible and the time to completion is measured and recorded as a performance variable. Thus, if a block design is used, the performance variable time is omitted by limiting the working time to e.g., 30 s. To better elicit the effects between TMT-A and TMT-B, it is necessary to control for the processing speed factor, since first, it is assumed that the blood oxygenation within a participant and TMT block is positively associated with the number of completed items. A second assumption is the positive association between blood oxygenation and task complexity. Normally in TMT-A more items are completed than in TMT-B (higher processing speed – higher O_2_Hb levels)^30,43^, whereas TMT-B, being the more difficult task (task complexity), also causes additional cortical activation. Consequently, both effects could obscure each other. This effect was confirmed in our previous study, where the results showed that the processing speed effects far outweighed the differences in task demands between TMTA and TMTB. From these two assumptions, we developed the correction method in our first study, which calculates the ratio of individual O_2_Hb levels per TMT block and averaged performance^19^.

Not surprisingly, the effect for the TMT-A versus TMT-B contrast found in the item-controlled analysis, was characterized by a higher hemodynamic response during TMT-B in comparison to TMT-A. The different performance requirements of the sub-tests TMT-A and TMT-B are thus shown on a neurophysiological level. Our findings are in line with the findings of several studies that agree on large-scale bilateral frontal activation, with higher values in the TMT-B than in TMT-A^47–49^. Other studies specified the region affected by a higher activity during TMT-B compared to TMT-A as the area of DLPFC^4,30,50^. In comparison to TMT-A, dual-task paradigms such as the TMT-B, where different tasks (number series and alphabet) have to be executed simultaneously, especially require executive functions as cognitive flexibility.

Contrary to our expectations, the item-corrected analysis showed no effect of age. This result was surprising as the older participants completed fewer items than the younger participants did and therefore the ratio calculation was suspected to reveal a higher activity for the older group. Interestingly, the blood oxygenation per item did not differ between the age groups, while the total blood oxygenation in the time-corrected data showed higher values within the older group in the ROIs of left DLPFC and right SAC. Consequently, higher O_2_Hb levels within the older participants in the time-corrected version could be interpreted as a compensation mechanism on the one hand, but on the other hand the validity of the statement must also be questioned, since time-correction in contrast to the item-correction does not take the individual performance into account. A more detailed investigation showed a linear negative correlation between age and number of completed items and a negative quadratic correlation between age and O_2_Hb levels in lDLPFC and rSAC. The quadratic relationship revealed a turning point at the age of 65–70 years, which was characterized by an increase of O_2_Hb levels up to 65–70 years and a subsequent decrease. Apart from the frequently used interpretation of increasing cortical blood oxygenation as age-related compensatory effect^28,30,31,51^, various other neuroscientific models, as the idea of age-related increase in O_2_Hb levels due to lower efficiency and specificity^52^, the dedifferentiation hypothesis^53^ and the CRUNCH-model^32^ contribute to the understanding of cognitive aging. It is worth highlighting the CRUNCH model, that assumes an additional recruitment of cortical resources with increasing task complexity until the age-dependent personal load limit is reached. However, the capacity of additional resources is limited and subsequently performance and brain activation drop again. What our data reveal is a decline in cortical activity starting at about age 65–70 years. It is thinkable that this age group already reaches their individual functional processing limit when performing TMT. Furthermore, the concept of neural dedifferentiation in the ageing brain also explains the results found, as age-related over-recruitment in the context of reduced performance has often been interpreted as a sign of neural dedifferentiation^54,55^. The assumption is based on the idea that older subjects, due to a lower specificity of brain regions, activate several functional areas together, which in younger subjects are specialized to only one cognitive functionality.

Interestingly, the analysis to control for medication (antiplatelets/anticoagulants/blood pressure medication) showed an effect in the item-corrected data, whereas the time-corrected results remained unaffected. Therefore, a direct influence of medication consumption on the fNIRS signal is unlikely. Nevertheless, note that only by adding the medication as a covariate, an age effect became apparent in the item-corrected data. The effect may be due to a positive influence of antihypertensives^56^ and anticoagulants^57^ on cognitive function, explaining a highlighting of age differences, as the older participants showed higher medication consumption compared to the younger group.

Finally, some limitations have to be considered.

First, our results raise the question of the limitation of our correction method. The decisive point is that the correction method introduced corrects for performance and since TMT performance is associated with age, it would also partially correct for age effects or, more precisely, for the part of the age-related variance in brain activity that is related to performance. With respect to the used correction method, one might argue that the correction of the O_2_Hb levels for the number of processed items consequently leads to higher blood oxygenation levels when participants show a reduced performance. However, this conclusion assumes that blood oxygenation would stay stable if volunteers work with less effort, which has been shown to not be the case^19^. On the one hand, the integration of processed items acts as efficiency measure, which is not considered in most other studies^4,30^. On the other hand, it could be argued that the ratio approach is too simplified to provide a well-founded result. However, we were able to demonstrate in our previous study^19^ that a correction of the fNIRS data by a regression approach (in which the average number of items was regressed out of the activity level for each individual at three different speed levels) and the simple ratio approach produced similar results. Since not every study can collect TMT data at three different speed levels, it provides a simple approximation. It should also be emphasized that our correction method is not intended to replace the original TMT measurement, which is not time-limited, nor to replace the time-corrected analysis for block designs. In case block designs are used, it is only intended to highlight performance differences as an additional index, which, as you can see from our results, should not be underestimated.

A second point to consider is the interpretation of the increase in hemodynamic response as a compensatory pattern. Although there is evidence in the literature for a compensatory nature of the increase in O_2_Hb levels in older subjects^28,58,59^, research varies greatly and the concept of age-related compensation was also critically questioned^60–62^. For example, Höller-Wallscheid et al.^60^ found an age-independent additional recruitment of cognitive resources from the contralateral hemisphere when participants where challenged by higher task demands, reflecting an adaptation to the increased requirements rather than a compensation for age deficits. Note that our results are only intended to represent one possible proposed interpretation. Other models of cognitive aging are also conceivable and have been discussed comparatively.

Third, despite many advantages (e. g. seated working, tests in realistic environment, social interaction…), the fNIRS emitters have a limited spatial resolution and a low penetration depth into the cranial calotte of 2–3 cm^63^. The near-infrared radiation therefore only detects superficial cortical structures and exhibits inter-individual variability depending on the path length and tissue composition of the grey matter.

The fourth limitation concerns the age range, since our study compared middle-aged and older participants. A comparison with a younger cohort (< 50 years) would probably have highlighted the results. However, it was precisely our concern to detect subtle differences in the incipient neurodegenerative processes, which we suspected to originate in the age of early retirement. One could criticize that the evaluation method we have chosen with separation of the cohort into two groups via a median split only represents a simplification. For this reason, we additionally checked the age effects in a continuous evaluation within the exploratory analysis.

**Figure 6:**
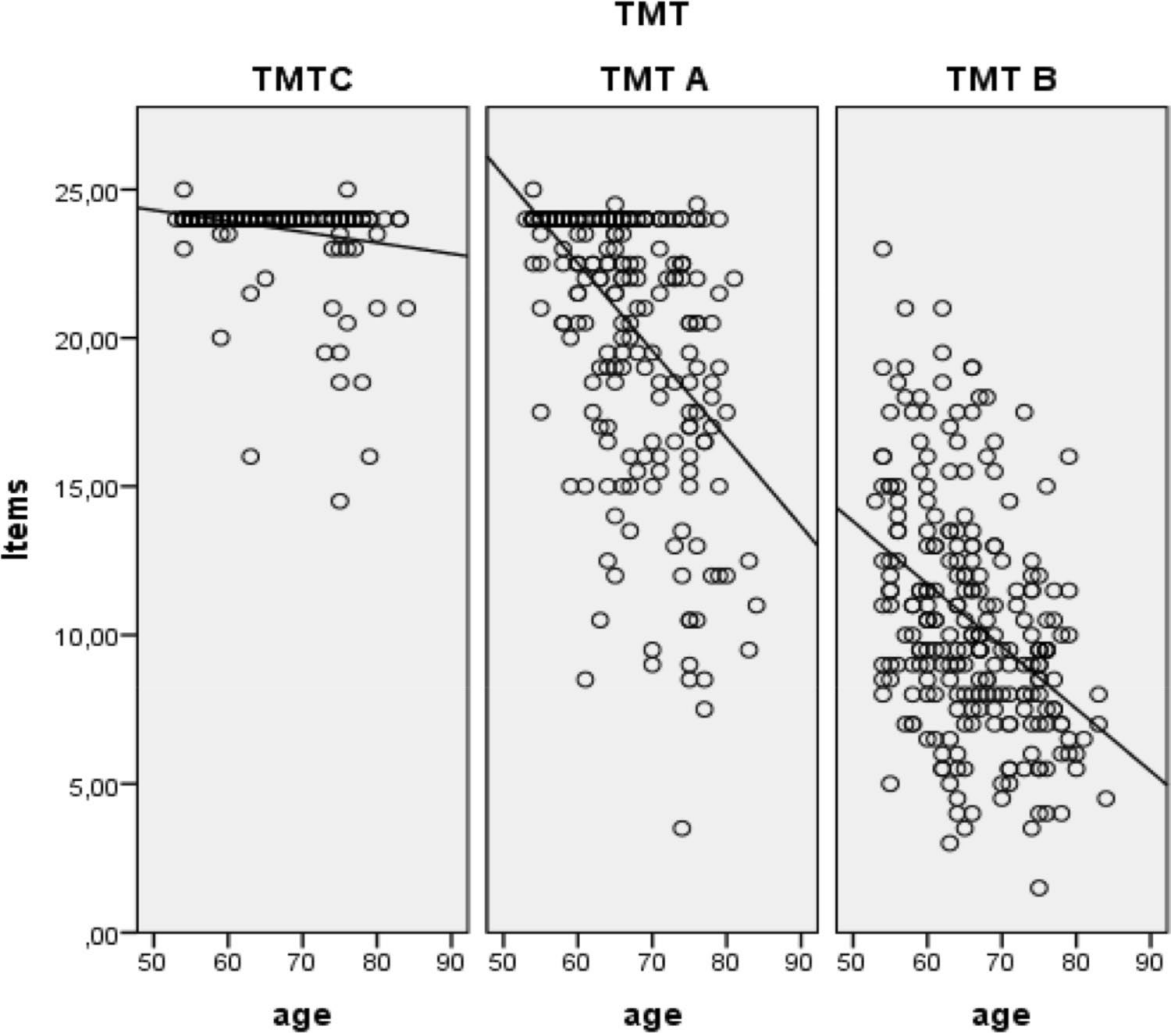
Negative linear relationship between age and number of completed items.

Another point that needs explanation is the high error variance visible on Figs. 5 and 6. This can be explained by an increased heterogeneity in performance, which is particularly evident in a high-aged group of participants. Furthermore, due to the tight schedule of the TREND study, the study at hand only includes a restricted TMT block number of two blocks per subject, which further increases the variability.

Finally, data were collected in a naturalistic observational study without examination of neurodegenerative marker such as amyloid-β or Tau in CSF or amyloid-β- or Tau-PET or MRI. For that reason, no assigning to a sub-group of participants with pre-clinical neurodegeneration was possible.

## Conclusions

To the authors' knowledge, this is the first study to examine the influence of age on TMT performance using a comparison of a time-correction and an item-correction method. To sum it up, our findings confirm several important age-related effects: fewer completed items, a lower error-rate during TMT-A and increased cortical activity in older participants. Our results emphasize the importance of applying a correction methods for the performance variable, since significant performance deficits exist within the older group. By slowing down the processing speed, deficits could be compensated according to a shift of the speed-accuracy trade-off. Contrary to our forecasts, the item-correction did not increase the sensitivity to age effects. Of particular importance, however, is the fact that the contrast between the TMT conditions TMT-A and TMT-B only became apparent after application of the correction method.

The ratio calculation can only be evaluated as a simplified approach to account for the individual performance and future research could aim to improve the correction method. Nonetheless, our comparison clearly shows that the number of processed items in TMT has a decisive influence on the overall capacity and should not be underestimated in the commonly used time-corrected designs.

## Data Availability

The datasets generated and analyzed during the current study are available from the corresponding author on reasonable request.

## References

[1] Rees P (2012). European regional populations: current trends, future pathways, and policy options. Eur. J. Popul..

[2] Alzheimer’s Disease International, World Alzheimer Report 2019: Attitudes to dementia. Alzheimer’s Disease International. retrieved from https://www.alz.co.uk/research/WorldAlzheimerReport2019-Summary.pdf (Accessed 5 Dec 2019), 2019.

[3] Fjell AM, Walhovd KB (2010). structural brain changes in aging: courses, causes and cognitive consequences. Rev. Neurosci..

[4] Hagen K (2014). Activation during the Trail Making Test measured with functional near-infrared spectroscopy in healthy elderly subjects. Neuroimage.

[5] Fjell AM (2014). What is normal in normal aging? Effects of aging, amyloid and Alzheimer's disease on the cerebral cortex and the hippocampus. Prog. Neurobiol..

[6] Walhovd, K.B., et al., Effects of age on volumes of cortex, white matter and subcortical structures. Neurobiol. Aging. 2005. **26**(9): 1261–70; discussion 1275–8. 10.1016/j.neurobiolaging.2005.05.020 16005549

[7] Courchesne E (2000). Normal brain development and aging: quantitative analysis at in vivo MR imaging in healthy volunteers. Radiology.

[8] Good CD (2001). A voxel-based morphometric study of ageing in 465 normal adult human brains. Neuroimage.

[9] Pfefferbaum A (1994). A quantitative magnetic resonance imaging study of changes in brain morphology from infancy to late adulthood. Arch Neurol.

[10] Morrison JH, Baxter MG (2012). The ageing cortical synapse: hallmarks and implications for cognitive decline. Nat. Rev. Neurosci..

[11] Hedden T, Yoon C (2006). Individual differences in executive processing predict susceptibility to interference in verbal working memory. Neuropsychology.

[12] Glisky EL (2007). Changes in cognitive function in human aging. Brain aging: Models, methods, and mechanisms.

[13] Colier WN, van Haaren NJ, Oeseburg B (1995). A comparative study of two near infrared spectrophotometers for the assessment of cerebral haemodynamics. Acta Anaesthesiol. Scand. Suppl..

[14] Ehlis AC (2014). Application of functional near-infrared spectroscopy in psychiatry. Neuroimage.

[15] Arbuthnott K, Frank J (2000). Trail making test, part B as a measure of executive control: validation using a set-switching paradigm. J. Clin. Exp. Neuropsychol..

[16] Stuss DT, Levine B (2002). Adult clinical neuropsychology: lessons from studies of the frontal lobes. Annu. Rev. Psychol..

[17] Spreen, O. and E. Strauss, A compendium of neuropsychological tests: Administration, norms, and commentary, 2nd ed. A compendium of neuropsychological tests: Administration, norms, and commentary, 2nd ed. 1998, New York, NY, US: Oxford University Press. xvi, 736-xvi, 736.

[18] Tischler, L. and F. Petermann, Trail making test (TMT). Vol. 58. 2010. 79–81.

[19] Rosenbaum, D., et al., Comparison of speed versus complexity effects on the hemodynamic response of the trail making test in block designs. Neurophotonics, 2018. **5**(4): p. 045007. 10.1117/1.NPh.5.4.045007 PMC6286664 30539043

[20] Tombaugh TN (2004). Trail Making Test A and B: normative data stratified by age and education. Arch. Clin. Neuropsychol..

[21] Mitrushina, M., et al., Handbook of normative data for neuropsychological assessment, 2nd ed. Handbook of normative data for neuropsychological assessment, 2nd ed. 2005, New York, NY, US: Oxford University Press. xxii, 1029-xxii, 1029.

[22] Hamdan AC, Hamdan EMLR (2009). Effects of age and education level on the trail making test in a healthy Brazilian sample. Psychol. Neurosci..

[23] Rasmusson DX (1998). Effects of age and dementia on the trail making test. Clin. Neuropsychol..

[24] Bäckman L (2004). Cognitive functioning in aging and dementia: the Kungsholmen project. Aging Neuropsychol. Cognit..

[25] Rodewald K (2012). Eine Normierungsstudie eines modifizierten Trail Making Tests im deutschsprachigen Raum. Zeitschrift für Neuropsychologie.

[26] Payer D (2006). Decreased neural specialization in old adults on a working memory task. NeuroReport.

[27] Cabeza R (2004). Task-independent and task-specific age effects on brain activity during working memory, visual attention and episodic retrieval. Cereb Cortex.

[28] Cabeza R (2002). Aging gracefully: compensatory brain activity in high-performing older adults. Neuroimage.

[29] Herrmann MJ (2006). Cerebral oxygenation changes in the prefrontal cortex: effects of age and gender. Neurobiol. Aging.

[30] Muller LD (2014). Neural correlates of a standardized version of the trail making test in young and elderly adults: a functional near-infrared spectroscopy study. Neuropsychologia.

[31] Rossi S (2004). Age-related functional changes of prefrontal cortex in long-term memory: a repetitive transcranial magnetic stimulation study. J. Neurosci..

[32] Reuter-Lorenz PA, Cappell KA (2008). Neurocognitive aging and the compensation hypothesis. Curr. Dir. Psychol. Sci..

[33] Li S-C, Lindenberger U, Sikström S (2001). Aging cognition: from neuromodulation to representation. Trends Cogn. Sci..

[34] Goh JO (2011). Functional dedifferentiation and altered connectivity in older adults: neural accounts of cognitive aging. Aging Dis..

[35] Li SC, Rieckmann A (2014). Neuromodulation and aging: implications of aging neuronal gain control on cognition. Curr. Opin. Neurobiol..

[36] Singh AK (2005). Spatial registration of multichannel multi-subject fNIRS data to MNI space without MRI. Neuroimage.

[37] Tsuzuki D (2007). Virtual spatial registration of stand-alone fNIRS data to MNI space. Neuroimage.

[38] Tsuzuki D, Dan I (2014). Spatial registration for functional near-infrared spectroscopy: from channel position on the scalp to cortical location in individual and group analyses. Neuroimage.

[39] Fishburn FA (2019). Temporal Derivative Distribution Repair (TDDR): A motion correction method for fNIRS. Neuroimage.

[40] Cui X, Bray S, Reiss AL (2010). Functional near infrared spectroscopy (NIRS) signal improvement based on negative correlation between oxygenated and deoxygenated hemoglobin dynamics. Neuroimage.

[41] Zhang X, Noah JA, Hirsch J (2016). Separation of the global and local components in functional near-infrared spectroscopy signals using principal component spatial filtering. Neurophotonics.

[42] Drane DL (2002). Demographic characteristics and normative observations for derived-trail making test indices. Neuropsychiatry Neuropsychol. Behav. Neurol..

[43] Gaudino EA, Geisler MW, Squires NK (1995). Construct validity in the trail making test: What makes part B harder?. J. Clin. Exp. Neuropsychol..

[44] Zimmerman, M.E., Speed–Accuracy Tradeoff, in Encyclopedia of Clinical Neuropsychology, J.S. Kreutzer, J. DeLuca, and B. Caplan, Editors. 2011, Springer New York: New York, NY. p. 2344–2344.

[45] Grady CL (2008). Cognitive neuroscience of aging. Ann N Y Acad Sci.

[46] Spreng RN, Wojtowicz M, Grady CL (2010). Reliable differences in brain activity between young and old adults: a quantitative meta-analysis across multiple cognitive domains. Neurosci. Biobehav. Rev..

[47] Shibuya-Tayoshi S (2007). Activation of the prefrontal cortex during the Trail-Making Test detected with multichannel near-infrared spectroscopy. Psychiatry Clin. Neurosci..

[48] Takeda C (2011). Identification of three factors influencing trail making test performance using multichannel near-infrared spectroscopy. Tohoku J. Exp. Med..

[49] Kubo M (2008). Increase in prefrontal cortex blood flow during the computer version trail making test. Neuropsychobiology.

[50] Jacobson SC (2011). An fMRI investigation of a novel analogue to the trail-making test. Brain Cogn..

[51] Reuter-Lorenz PA (2000). Age differences in the frontal lateralization of verbal and spatial working memory revealed by PET. J. Cogn. Neurosci..

[52] Morcom AM, Henson RNA (2018). Increased prefrontal activity with aging reflects nonspecific neural responses rather than compensation. J. Neurosci..

[53] Lindenberger U, Baltes PB (1997). Intellectual functioning in old and very old age: cross-sectional results from the Berlin aging study. Psychol. Aging.

[54] Morcom AM, Li J, Rugg MD (2007). Age effects on the neural correlates of episodic retrieval: increased cortical recruitment with matched performance. Cereb Cortex.

[55] de Chastelaine M (2011). The effects of age, memory performance, and callosal integrity on the neural correlates of successful associative encoding. Cereb Cortex.

[56] Levi Marpillat N (2013). Antihypertensive classes, cognitive decline and incidence of dementia: a network meta-analysis. J. Hypertens.

[57] Friberg L, Rosenqvist M (2018). Less dementia with oral anticoagulation in atrial fibrillation. Eur. Heart J..

[58] Du Y (2016). Increased activity in frontal motor cortex compensates impaired speech perception in older adults. Nat. Commun..

[59] Grady CL, McIntosh AR, Craik FIM (2005). Task-related activity in prefrontal cortex and its relation to recognition memory performance in young and old adults. Neuropsychologia.

[60] Höller-Wallscheid, M.S., et al., Bilateral recruitment of prefrontal cortex in working memory is associated with task demand but not with age. Proceedings of the National Academy of Sciences, 2017: p. 201601983. 10.1073/pnas.1601983114 PMC5293052 28096364

[61] Colcombe SJ (2005). The implications of cortical recruitment and brain morphology for individual differences in inhibitory function in aging humans. Psychol. Aging.

[62] Duverne S, Motamedinia S, Rugg MD (2009). The relationship between aging, performance, and the neural correlates of successful memory encoding. Cereb Cortex.

[63] Haeussinger FB (2011). Simulation of near-infrared light absorption considering individual head and prefrontal cortex anatomy: implications for optical neuroimaging. PLoS ONE.

